# “This does not sound like me”? Vocal identity negotiation in one-to-one voice teaching

**DOI:** 10.3389/fpsyg.2026.1928649

**Published:** 2026-08-25

**Authors:** Congzhi Lin

**Affiliations:** School of Music, The Ohio State University, Columbus, OH, United States

**Keywords:** music student, singing, singing experiences, vocal identity, voice change

## Abstract

**Background:**

In one-to-one voice teaching, teachers frequently evaluate and demand alterations toward idealized vocal targets. However, there is a paucity of research exploring the identity negotiation processes students undergo when exposed to unfamiliar vocal productions.

**Aims/Objectives:**

This qualitative study explores students' understanding of the teacher-prescribed “correct voice” and traces their identity negotiation trajectory from their original voice to the mandated vocal output.

**Methods:**

Utilizing a qualitative approach, in-depth data were collected from vocal students and analyzed thematically.

**Results:**

Four overarching themes emerged: *Recognition of Teacher Authority, Perceived Uncertainty, Vocal Value Construction*, and *Vocal Agency*. Students initially legitimize the unfamiliar voice based on teacher authority, with their trust or doubt mediating their vocal perception. Experiencing the unfamiliar voice continuously triggers cognitive, emotional, and competence-based uncertainties. However, this uncertainty stimulates crucial reflection between their inherent voice and the required voice. Through sustained engagement and reflective learning, students reconstruct the technical, aesthetic, emotional, and social values of the new voice, ultimately cultivating vocal agency through independent evaluative standards and self-regulated strategies.

**Conclusions:**

Developing vocal agency represents a successful outcome of identity negotiation. These insights provide significant implications for guiding the psychological aspects of music teaching, ultimately promoting learner autonomy and wellbeing in vocal education.

## 1 Introduction

In one-to-one voice teaching, teachers frequently guide students toward an idealized, “correct” vocal sound through demonstrations, evaluative feedback, and technical explanations. However, this “correct” voice is rarely a fixed, transparent, or universally natural standard (Hausknecht et al., 2024; Vurma and Ross, 2002); rather, it is heavily mediated by pedagogical traditions, subjective aesthetic preferences, and the teacher's own embodied experiences (Frič and Pavlechová, 2020; Vurma and Ross, 2002; Brown, 2025; Mitchell, 2014). Consequently, the extensive terminology utilized to define these professional vocal targets is often characterized by a conspicuous lack of scientific standardization. For instance, Hausknecht et al. (2024) identified 292 distinct evaluative terms across vocal syllabi, noting a 61% redundancy rate and a profound inconsistency in application. Contemporary research further argues that vocal terminology classifications often fail to capture the highly nuanced and diverse characteristics of individual timbres (Frič and Pavlechová, 2020).

Because singing is a profoundly sensorimotor discipline, educators frequently rely on subjective assessments, metaphorical language, and imagery-based pedagogy to convey these abstract vocal standards (Morales-Villar et al., 2023; Brown, 2025; Mitchell, 2014; Ragan, 2018). While figurative language can facilitate experiential learning, it often obscures underlying physiological principles (Gill and Herbst, 2016; Lillis, 2021). Spatial concepts such as “forward” or “backward” placement, for example, are frequently misinterpreted; empirical evidence shows that 46% of students fail to accurately identify the intended vocal placement, and 11% judge it as the exact opposite (Vurma and Ross, 2002). This over-reliance on ambiguous metaphors creates a critical perceptual gap between a teacher's descriptive terminology and a student's unique somatic sensations, potentially leading to misinterpretation, stifled vocal potential, or even unhealthy phonatory habits (Carroll et al., 2006; Ware, 2013; Henrich et al., 2008).

Navigating this ambiguous pedagogical landscape relies also heavily on the teacher-student relationship and the nature of the evaluative feedback provided (Bonneville-Roussy et al., 2020; de Bruin, 2024; Fletcher et al., 2024; Gaunt, 2010; Hogle, 2021; Johansson, 2013; Lewis and Hendricks, 2022; McPherson and Blackwell, 2025; Blackwell et al., 2025). Because the human voice is inextricably linked to the self, singers often experience unique psychological vulnerabilities (Serra-Dawa, 2015; Sweet, 2018). When pedagogical directives are perceived as unclear, restrictive, or disconnected from students' embodied experiences, they may encounter vocal inhibition, self-doubt, anxiety, and a diminished sense of vocal worth (Krause et al., 2025; Lewis and Hendricks, 2022; Gaunt, 2011; O'Bryan, 2015; Sweet, 2018). Conversely, autonomy-supportive environments grounded in relational trust allow students to articulate confusion without fear, enabling teachers to translate abstract evaluations into comprehensible, tailored goals (Fletcher et al., 2024; Blackwell et al., 2025). Effective vocal instructors should foster students' independence and provide autonomy support, nurture singers' creativity, and demonstrate an intuitive understanding of students' various challenges throughout the learning and teaching experience (Chapman, 2006; Bonneville-Roussy et al., 2020).

Ultimately, the trajectory from a student's habitual voice to the teacher-prescribed ideal extends far beyond the mere acquisition of technical skills; it is a profound process of vocal identity negotiation. Through somatic experiences, continuous self-monitoring, and dynamic interactions with their teachers, emerging singers construct and renegotiate their understanding of “who they are as vocal performers” (MacDonald et al., 2002; O'Bryan, 2015; Després and Dubé, 2020; Forbes et al., 2025). The trajectory from initial vocal instruction to the attainment of teacher-prescribed standards is heavily mediated by embodied cognition—encompassing somatic awareness, kinesthetic engagement, and sensory feedback—to optimize musical performance (Jiang and Wu, 2025). Tensions frequently emerge when the teacher-endorsed vocal ideal conflicts with the student's existing sense of vocal selfhood, prompting cognitive dissonance encapsulated in reflections such as “this does not sound like me.” In such liminal spaces, students must continuously evaluate and internalize these external standards to align their physiological output with their internalized self-perception (Crow et al., 2021). The ultimate echelon of this vocal praxis is for learners to transition from relying on didactic instruction to achieving vocal autonomy and self-regulation (Rudd et al., 2006). However, numerous studies indicate that music teachers primarily employ controlling feedback and modeling in vocal training (McPherson and Blackwell, 2025; Crocco et al., 2020; Bonneville-Roussy et al., 2020), with comparatively less focus on perceptual training and guiding students to self-monitor and regulate their performance and motivation (Crocco et al., 2020; McPherson and Blackwell, 2025).

Despite increasing recognition of the social and embodied dimensions of voice learning (O'Bryan, 2015; Monks, 2003; Després and Dubé, 2020; Lewis and Hendricks, 2022; Forbes et al., 2025; Sweet and Parker, 2019; Hogle, 2021; Chong et al., 2024; Monks, 2003; Després and Dubé, 2020; Forbes et al., 2025), existing scholarship has predominantly adopted a teacher-centric lens. Research has extensively documented instructional methodologies or the unidirectional influence of educators on identity formation, yet scant attention has been paid to the student's subjective perspective (Crow et al., 2021). Little is known about the internal mechanisms through which students receive, internalize, and negotiate the tensions between pedagogically prescribed ideals and their authentic voices within one-to-one settings. While the authoritative nature of one-on-one studio instruction can build crucial trust, an over-reliance on a single pedagogue's subjective judgment risks undermining the student's autonomy, shifting their motivation from intrinsic artistic progress to the mere pursuit of external validation (Gaunt, 2010; Blackwell et al., 2025).

To address this gap and align with the contemporary paradigm shift toward student-centered music education (Li et al., 2026; Lã, 2017), this qualitative study conceptualizes vocal learning as an active identity negotiation process. By prioritizing the student's subjective agency, the twofold aim of this research is to empower learners in developing autonomous evaluative judgment, and to elucidate the cognitive and emotional perplexities they experience during dyadic vocal learning, thereby equipping vocal educators with empirical insights for implementing more individualized and empathetic pedagogical strategies. Accordingly, this study addresses the following research questions:

1. How do students understand and perceive the “correct voice” required by their teachers in one-to-one voice teaching?
2. How do students negotiate their vocal identities by interpreting and evaluating the relationship between their existing vocal experiences and the professional vocal standards expected by their teachers?
3. How does this process of identity negotiation influence students' subsequent learning behaviors, responses to vocal training, and the development of their vocal identities?

## 2 Materials and methods

### 2.1 Data collection: semi-structured interviews

Semi-structured interviews were employed as the primary method of data collection. This approach was selected because it is both practically feasible within the educational contexts of conservatories and university music departments and capable of generating rich qualitative evidence regarding students' cognitive experiences. Compared with more structured forms of interviewing, semi-structured interviews allow researchers to explore participants' individual experiences in greater depth while maintaining consistency across core research topics.

Furthermore, one-to-one interviews can be conducted without disrupting institutional teaching arrangements or creating concerns among faculty members and students. An informant-centered interviewing style facilitates rapport between the interviewer and participants, thereby enhancing the authenticity of participants' accounts of their learning experiences and cognitive constructions (Powney and Watts, 1987; Cooper, 1993).

The researcher has more than 10 years of experience in one-to-one vocal training, providing a shared frame of reference with participants throughout the interview process. Although such familiarity may introduce potential researcher bias, an awareness of this shared background also enabled the interviewer to probe more deeply into participants' perceptions, interpretations, and reflections concerning their vocal learning experiences. Throughout the study, reflexivity was maintained to minimize the influence of the researcher's prior experiences on data interpretation.

All participants were undergraduate or postgraduate vocal performance students enrolled in public conservatories or comprehensive universities in China. Participants represented three major vocal specializations: Western classical singing (Bel Canto), Chinese national singing, and popular singing. Each participant received at least 1 h of individual vocal instruction per week. Their institutions employed a studio-based model of one-to-one vocal teaching, in which each student worked with a designated vocal instructor over an extended period. This highly individualized instructional setting and stable teacher–student relationship provided an appropriate context for examining students' understandings of the “correct voice” and the development of their vocal identities.

A total of 20 participants (S1–S20), aged between 21 and 32 years, were recruited for this study. Participants were selected through a combination of purposive sampling and snowball sampling (see Table 1). Recruitment announcements describing the research purpose, eligibility criteria, and interview procedures were posted on two widely used Chinese social media platforms, Xiaohongshu and Douyin, between 28 May and 9 June 2026. Sixteen participants voluntarily contacted the researcher via private messages after viewing the recruitment advertisements. The remaining four participants were recruited through recommendations from the researcher's colleagues and peers and were subsequently included after meeting the inclusion criteria, following a snowball sampling strategy.

**Table 1:** Participant information.

ID	Age	Institution	Degree	Currently enrolled	Voice type	Singing style	Years of vocal training
S1	25	Fujian Normal University	Master	Yes	Soprano	National singing	7
S2	24	Beijing Normal University	Master	Yes	Soprano	National singing	8
S3	25	Xinjiang Arts University	Master	Yes	Mezzo-soprano	Bel canto	10
S4	26	Yonsei University, South Korea	Master	No	Tenor	Bel canto	9
S5	24	Shandong Normal University	Master	Yes	Baritone	Bel canto	6
S6	21	China Conservatory of Music	Bachelor	Yes	Soprano	National singing	5
S7	24	Inner Mongolia Arts University	Master	Yes	Soprano	Bel canto	10
S8	25	Shanghai Conservatory of Music	Master	Yes	Tenor	Bel canto	8
S9	22	Zhejiang Conservatory of Music	Bachelor	Yes	Tenor	Popular singing	4
S10	25	Zhejiang Conservatory of Music	Master	Yes	Soprano	Bel canto	10
S11	24	Central China Normal University	Master	Yes	Soprano	Bel canto	7
S12	24	Xinjiang Arts University	Master	Yes	Soprano	National singing	8
S13	30	The Rimsky-Korsakov St. Petersburg State Conservatory, Russia	Doctoral	No	Tenor	Bel canto	12
S14	32	Chung-Ang University, South Korea	Doctoral	No	Tenor	Bel canto	14
S15	21	Jimei University	Bachelor	Yes	Tenor	Bel canto	3
S16	27	Boston University, USA	Doctoral	No	Baritone	Bel canto	10
S17	23	Xiamen University	Bachelor	Yes	Soprano	Bel canto	5
S18	25	Beijing Normal University	Master	Yes	Soprano	Bel canto	7
S19	25	Jāzeps Vitols Latvian Academy of Music, Latvia	Master	Yes	Countertenor	Bel canto	8
S20	25	Xiamen University	Bachelor	No	Baritone	Bel canto	6

The interview protocol was developed by the researcher and organized around four broad areas of inquiry: (1) students' understandings and perceptions of the “correct voice”; (2) the circumstances under which feelings of vocal unfamiliarity emerged; (3) the processes through which students negotiated their vocal identities; and (4) the influence of identity negotiation on subsequent responses to vocal training. Rather than imposing a fixed sequence of questions, the interview guide was designed to encourage participants to articulate their own experiences, concerns, and interpretations within these overarching domains. To facilitate deeper exploration when appropriate, a series of follow-up prompts was prepared in advance and used flexibly according to the direction of each interview.

All interviews were conducted individually via online video conferencing platforms. With participants' informed consent, each interview was audio-recorded and lasted no less than 50 min. Following data collection, the recordings were transcribed verbatim by the researcher, yielding approximately 138,700 Chinese characters. Each transcript was subsequently returned to the corresponding participant for member checking, allowing participants to review, revise, or clarify their responses. Only transcripts approved by participants were included in the final dataset for analysis.

### 2.2 Ethical considerations

Participation in this study was entirely voluntary. During the initial recruitment process, the researcher explained the aims and procedures of the study to all prospective participants. Participants were explicitly informed that their vocal instructors would not be involved in any stage of the research to minimize potential concerns regarding teacher–student relationships and to encourage candid responses. Prior to the interviews, participants received a written information sheet outlining the research background, objectives, and methodological procedures.

Participants were assured that they would have the opportunity to review and amend their interview transcripts before the data were analyzed. They were further informed that declining to participate or withdrawing from the study would have no influence on their academic standing, access to instructional resources, or assessment within their institutions. Prior to each interview, participants received a full explanation of the study and provided verbal informed consent, in accordance with the protocol approved by the Ethics Committee of Nanjing Normal University (Approval No. NNU202603017); interviews were audio-recorded only with participants' explicit permission. After participants had reviewed and, where necessary, revised their transcripts, verbal informed consent was reconfirmed to ensure participants' continued agreement for the use of the finalized data in academic analysis and publication. In addition, the researcher discussed the potential risks and benefits of participation with each participant to ensure that consent was fully informed.

### 2.3 Data analysis

The present study sought to generate empirical evidence concerning students' vocal identity negotiation within one-to-one voice teaching rather than relying solely on theoretical discussion. Interview data were analyzed using thematic analysis as proposed by Braun and Clarke (2006). Thematic analysis is a flexible yet systematic qualitative analytic approach that enables researchers to identify, analyze, and interpret meaningful patterns across a dataset. It is particularly well suited to exploring how individuals make sense of their lived experiences, making it highly appropriate for addressing the research questions of this study.

The analysis followed Braun and Clarke's (2006) six-phase framework and was supported by NVivo software for data management and coding. First, the researcher repeatedly read all interview transcripts to become thoroughly familiar with the dataset while recording preliminary analytic reflections. Second, the transcripts were systematically coded in NVivo to generate initial codes relevant to the research questions. Third, conceptually related codes were grouped into candidate themes. These themes were then iteratively reviewed, refined, and compared against the dataset to ensure coherence within themes and clear distinctions between themes. Finally, each theme was defined and named, and the analytical findings were developed through close integration of thematic interpretations with representative participant quotations. The complete coding is presented in Table 2.

**Table 2:** Thematic coding results.

Themes	Codes	Initial codes
Recognition of teacher authority	Teacher personal qualities	Sense of responsibility; stubbornness; friendliness (encouragement, praise, respect); own vocal quality; personal charisma; patience; inclusiveness
	Teacher professionalism	Clarity of explanation; theoretical instruction (conveying theory, conceptual framing, cultivating aesthetic standards, use of technical terminology); Individualized instruction; integration of multiple methods; teacher intervention; classroom standards; student-centeredness; clarity of goals
	Teaching techniques	Progressive/repetitive training (graduated exercises, exaggerated exercises, fundamental vocalizes, high repetition, muscle memory); physical relaxation (yawning to relax the throat, throat adjustment, bodily gestures, tongue-root relaxation); vocal placement training (voice placement, resonance expansion, humming for placement, direction of sound, breath exercises, breath support);
	Teaching approach	repertoire-oriented adjustment (adjusting the voice according to the piece being performed); goal-oriented training (vocal uniformity, approximating the ideal voice); text/language analysis (lyric analysis, diction refinement); demonstration (modeling, recording imitation, teacher–student unison singing, imitating the teacher's voice); tool-assisted instruction (note-taking, balance ball, straw phonation)
	Teacher–student relationship	Doubt; trust; estrangement; dependency
Perceived uncertainty	Emotional turbulence to strange voice	Confusion, unfamiliarity, anxiety, joy, disorientation, novelty, non-acceptance, discomfort, comfort, doubt, excitement, disappointment, surprise, fear, numbness; frustration
	Competence mismatch and self-doubt	Uncertainty in vocal judgment; uncertainty in vocal choice; lack of future planning; loss of patience with singing; difficulty changing one's vocal concept; fear of imitating others
	Difficulty in skill internalization	Technical instability; difficulty internalizing skills; mismatch between technical demands and ability; lack of a clear concept of the “correct voice”; instability in judging the “correct voice”; insufficient aptitude for vocal learning; instability of vocal state; a lag in conceptual understanding; self-doubt
	Surface compliance	Anxiety about teacher evaluation; worry over negative feedback; perceived risk in self-expression; apprehension about vocalizing in class
Vocal value construction	Technical	Continuous practice; exploring the unfamiliar voice; improvement in singing technique
	Aesthetic	Awareness of aesthetic diversity (differences across ethnicity/nation/custom; individual differences; age differences); aesthetic openness (accepting diverse vocal styles; moving beyond a single vocal standard; expanding the boundaries of vocal aesthetics); awareness of musical expression (imagination; artistic interpretation); timbre perception (age-appropriate; voice-type-appropriate; expressive of inner feeling; clear; full; brightness; healthy; even; clean; emotionally controlled; comfortable; unified; projection; flexible; pleasant; sincere; natural)
	Cognitive	Confusion deepening understanding; challenging prior conceptions; active inquiry; consulting multiple teachers' evaluations; reflective learning; self-directed exploration
	Emotional	Acceptance of the new voice (identification, reduced resistance, continued practice); emotional adaptation (sense of comfort, stability)
	Social	Positive teacher feedback; peer recognition; positive audience evaluation
Vocal agency	Judgment	Multidimensional judgment criteria (integrating auditory and somatic feedback; accounting for environmental and contextual factors; aligning with repertoire-style demands; synthesizing multiple sources of evaluative information); development of independent evaluative ability (moving beyond reliance on a single authority's judgment; reduced dependence on external evaluation; establishing personal judgment standards); muscular sensation (muscular sensation as a basis for judgment; prioritizing bodily feedback; forming embodied experiential knowledge)
	Competence	Contextualized vocal choices (adjusting the voice according to repertoire demands, emotional content, or performance goals); self-monitoring and self-regulation (actively observing one's physical state; autonomously adjusting vocal strategies; monitoring changes in vocal outcome); physical comfort (attending to bodily sensation; prioritizing sustainable vocal production; avoiding excessive reliance on technique)
	Self-efficacy	Alignment with future career plans; development of self-directed learning

To enhance the trustworthiness of the study, issues of credibility, dependability, and transferability were considered throughout the research process (Cohen et al., 2000). Credibility was strengthened through several complementary strategies. First, participants were recruited through a combination of purposive and snowball sampling to ensure diversity in institutional backgrounds and educational levels. Second, in-depth semi-structured interviews generated rich and contextually grounded accounts of participants' experiences. Third, member checking was employed by inviting participants to review and verify their interview transcripts, thereby ensuring that the researcher's interpretations accurately reflected participants' intended meanings. Dependability was addressed by systematically applying Braun and Clarke's (2006) six-phase framework in a consistent and transparent manner, with each stage of coding and theme development documented within NVivo to allow the analytic process to be traced. Transferability was supported through the provision of detailed contextual information regarding participants' institutional backgrounds, educational levels, vocal genres, and training experience (see Table 1), allowing readers to judge the extent to which the findings may apply to other, similar educational settings.

The researcher possesses more than 10 years of formal vocal training and holds a doctoral qualification in vocal performance, providing a shared professional frame of reference with participants. While this insider perspective facilitated rapport and encouraged rich, nuanced accounts of participants' experiences, it also presented the possibility of interpretive bias. To address this issue, reflexivity was maintained throughout the thematic analysis process. The researcher continuously examined how personal assumptions, disciplinary knowledge, and prior experiences might shape data interpretation, thereby reducing the influence of subjective bias and enhancing the overall trustworthiness of the findings.

## 3 Results

### 3.1 Recognition of teacher authority: compliance, dependency, and skepticism

The data reveal that students' initial willingness to experiment with unfamiliar vocal productions stems not from an intrinsic identification with the sound itself, but rather from compliance with teacher authority. This compliance is fundamentally predicated on the students' trust in the teacher's professional competence. Multiple participants indicated that their belief in the teacher's expertise and aesthetic judgment served as the psychological bedrock that sustained their efforts to produce the mandated unfamiliar voice.

This compliance is operationalized through how students receive and process the teacher's criteria for a “correct voice.” Participants universally reported that teachers predominantly employ three pedagogical modalities: vocal modeling (demonstration), embodied guidance, and linguistic description. As S2 elucidated:

“*During lessons, the teacher usually demonstrates by singing first. But when my voice fails to meet the requirement, the teacher resorts to abstract metaphors, like imagining the sound floating above my head, or sometimes directly adjusts my jaw or abdomen to assist my phonation.”* (S2)

Vocal modeling, particularly singing demonstrations, emerged as the most direct and frequently cited pedagogical tool, explicitly mentioned by 19 participants (nearly the entire sample). Teachers utilize modeling to provide an auditory paradigm of the idealized voice, subsequently demanding imitation:

“*Vocal modeling is crucial in vocal learning. The teacher must sing it out for the student to comprehend what kind of sound is desired.” (S3)*

“*My teacher uses terminology like ‘vocal fold closure' or ‘resonance placement,' but also personally demonstrates for me to imitate the sensation. To me, modeling is the most transparent instructional method.” (S17)*

Occurring with almost equal frequency is embodied guidance—interventions involving breath management, resonance placement, and somatosensory regulation. This modality attempts to bypass the limitations of pure linguistic description by directly mobilizing the student's kinesthetic awareness. For instance, S16 noted, “When my larynx rises, the teacher instructs me to step one leg forward and shift my center of gravity to help stabilize the laryngeal position.” Similarly, S2 described a somatic approach utilizing humming: “A method my teacher frequently uses is humming—first opening the nasal and head cavities via breath, then forming a stable resonant space in the head, ensuring the voice remains in a high placement.”

In stark contrast, linguistic description was identified as the primary source of cognitive dissonance and pedagogical friction. Numerous participants explicitly stated that within the one-on-one studio, they struggled to decipher abstract vocal metaphors and specialized terminology, rendering them unable to fulfill the teacher's expectations:

“*Initially, my teacher used highly abstract language to describe the voice, like ‘let the sound emerge from between your eyebrows,' which left me profoundly confused.” (S13)*

“*Vocal learning requires imagination. When the teacher says ‘let the sound travel upwards,' without imagination, I am completely clueless about what they mean.” (S3)*

“*In the early stages, the teacher used professional terms like ‘vocal fold closure,' and I had absolutely no idea how to execute that physically.” (S11)*

The interview data indicate that low-intervention, heuristic pedagogical approaches—where students are permitted to freely explore—are exceedingly rare. For most participants, acquiring the “correct voice” is not a solitary, reflective process. Instead, they are subjected to a relentless succession of teacher demonstrations, embodied cues, and linguistic prompts, leaving minimal space for independent exploration devoid of immediate teacher intervention. This highly prescriptive instructional scaffolding inherently demands that students temporarily suspend their independent judgment at the onset of the negotiation process, submitting to the teacher's directives to proceed. In the context of dyadic vocal instruction, pedagogical compliance is not merely the most economical choice; it is the mechanism through which students legitimize their continued experimentation and learning.

For some participants, this temporary suspension of personal judgment calcifies into chronic dependency. Several students admitted to lacking a coherent conceptualization of the “correct voice,” while others described a state of cognitive lag. In the absence of an internalized evaluative framework, teacher feedback and modeling naturally become the sole reliable references. Rather than an active choice, relying on the teacher becomes the only viable option when the student's own judgmental scaffolding remains unformed.

“*I genuinely cannot hear whether I am producing the correct sound, because there is a massive discrepancy between the sound I hear directly and the sound in a recording. Therefore, I heavily rely on my teacher's judgment.” (S16)*

“*I struggle to properly digest the knowledge imparted by my teacher, and I've learned such a mishmash of techniques that they feel like they are fighting within my body. Consequently, I choose to trust the teacher's judgment.” (S1)*

However, compliance and dependency are not the sole responses; a significant proportion of participants simultaneously expressed skepticism toward pedagogical methods or teacher judgments, particularly concerning aesthetic divergences and voice type classification (Fach). Multiple participants cited aesthetic misalignments with their teachers or felt their innate voice type contradicted the teacher's diagnosis. For example, S10 recounted a disorienting experience where their first teacher classified them as a mezzo-soprano, whereas the second categorized them as a soprano, leading to chronic confusion regarding their vocal identity. Crucially, skepticism toward the teacher directly mediates the student's acceptance of the unfamiliar voice. The deeper the doubt regarding the teacher's judgment, the less likely the student is to authentically internalize the mandated new voice, even if they maintain superficial compliance during practice.

### 3.2 Experiencing uncertainty through encountering unfamiliar voices

Regardless of whether students hold attitudes of trust, dependency, or latent skepticism toward teacher authority, they must inevitably engage in repeated experimentation with the mandated unfamiliar voice. Both within the studio and during solitary practice, they are forced to independently confront the inherent ambiguity of this new vocal production. This multidimensional uncertainty marks the onset of a profound identity-level conflict: as noted in the theoretical framework, the unfamiliar voice disrupted students' existing assumptions about singing and challenged their sense of competence, certainty, and self-understanding.

The conflict of vocal identity first manifests as cognitive uncertainty. Students frequently lack a clear conceptualization of the “correct voice” and possess no internalized criteria for evaluating it. The etiology of this cognitive dissonance is twofold: firstly, the linguistic ambiguity and metaphorical nature of pedagogical terminology render it difficult for students to form precise evaluative standards; secondly, students who over-rely on teacher judgments inherently lack an independent cognitive framework, meaning their epistemological uncertainty is never truly resolved. Intertwined with this cognitive ambiguity is a profound competence-related uncertainty. Several participants reported a chronic inability to master the prescribed vocal techniques or described their vocal state as highly volatile, highlighting a stark discrepancy between pedagogical expectations and their current physiological capabilities:

“*When I first started vocal training, my voice would ‘regress to its habitual state' the moment I stepped out of the studio. My technique was highly unstable; conceptually, I knew what I was supposed to do, but physiologically, I simply couldn't execute it.” (S5)*

“*I can achieve a great vocal state during the lesson, but when I practice alone, I completely lose that sensation. It remains this way even now. Ultimately, my technique is volatile, and my foundational skills require consolidation.” (S12)*

The confrontation with an unfamiliar voice is intrinsically coupled with intense emotional responses. For the majority of participants, the initial encounter with the new sound precipitated an indescribable somatic discomfort—a feeling that the voice did not sound like “them,” frequently triggering acute self-doubt. Expressions of confusion, skepticism, and anxiety permeated the narratives detailing these initial encounters. Notably, 12 participants explicitly stated they rejected the new sound they produced, while 11 expressed profound somatic and psychological maladaptation to it.

“*That sound was entirely different from how I sang in high school. It didn't sound like me at all. I was deeply resistant and felt I shouldn't be producing such an awful sound.” (S12)*

“*The reason I rejected the new voice was my conviction that this pedagogical method was incompatible with my current vocal condition and my specific voice type (Fach).” (S15)*

“*The new voice felt deeply alien to me. It was as if my body hadn't yet adapted to this phonatory mechanism; I experienced profound somatic unfamiliarity.” (S11)*

“*I felt the new sound was too folksy or lacked resonance. I vehemently disliked it. I was bewildered and constantly doubted whether the sound I was producing was actually ‘correct'.” (S16)*

While this emotional turbulence often precipitated a transient destabilization of their self-efficacy and identity, not all affective experiences were strictly negative. A subset of participants reported feelings of novelty, anticipation, and even excitement upon encountering the unfamiliar voice. Although such positive affect was significantly less frequent than negative responses, its presence underscores that the negotiation of the unfamiliar voice generates a highly complex, heterogeneous spectrum of emotional experiences:

“*My immediate reaction was sheer novelty; I was astonished that I was even capable of producing such a sound.” (S13)*

“*At that time, I felt curious and even exhilarated. I just kept experimenting using the teacher's methods; I am not averse to any new vocal techniques or sounds.” (S17)*

Beyond cognitive and emotional challenges, students navigated significant relational uncertainty. They lacked clear judgment on whether—and how—they should voice their dissent regarding the teacher's aesthetic or technical directives. This relational precarity differs fundamentally from pedagogical skepticism: while skepticism points to a disagreement with a specific technical judgment, relational uncertainty points to the interpersonal dilemma of how to maneuver within the power dynamics once that disagreement arises. Consequently, surface compliance (or performative compliance) emerged as a prevalent covert coping strategy. Students would performatively execute the teacher's demands within the studio, yet privately retain their own judgments, never authentically internalizing the mandated standards.

“*Faced with confusion, I rarely dared to question my teacher. He is extremely strict, and I simply lacked the courage to challenge him.” (S4)*

“*When the teacher asked me to produce the new sound, I felt I could achieve the repertoire using my old methods. Therefore, I didn't genuinely attempt his approach; I just performatively cooperated during the lesson to get by.” (S13)*

“*In the studio, I followed his methods and cooperated as much as possible, but during my private practice, I completely abandoned his approach.” (S15)*

The intricate intertwining of cognitive, competence-based, emotional, and relational uncertainties constitutes the core internal lived experience during the incipient phases of identity negotiation. Crucially, however, uncertainty does not signify the termination of the negotiation process; rather, it frequently acts as a catalyst for transformation. For a substantial cohort of participants, the negotiation process resembled a continuous cycle of heuristic probing and recalibration. The onset of confusion prompted proactive exploration and the testing of novel vocal mechanisms. Fourteen participants explicitly noted that their encounter with the unfamiliar voice initiated sustained, exploratory practice, with some reframing the confusion itself as the primary engine for their vocal advancement.

“*I didn't find it strange at all; rather, I couldn't wait to explore this novel sound. I love producing diverse sounds and practicing relentlessly until I can master them fluently.” (S3)*

“*Honestly, I experienced significant oscillation and wavering when faced with the new voice. But I discovered that this very oscillation fostered my growth. It compelled me to contemplate the myriad possibilities of the human voice, ultimately allowing me to master my own vocal instrument with far greater conscious agency.” (S17)*

### 3.3 Constructing the value of the unfamiliar voice

Amidst the aforementioned uncertainty, students who cultivated a heuristic desire for exploration began to initiate profound self-inquiry during the negotiation phase: “Is this my voice?”, “Why must I adopt this sound?”, and ultimately, “What is my true voice?” The resolution of these ontological questions stems not from passive subjugation to teacher authority, but rather from the students' active, gradual construction of value around the unfamiliar voice. This value construction is not a unidimensional, linear accumulation; rather, it unfolds simultaneously across five distinct yet intersecting dimensions—technical, aesthetic, cognitive, emotional, and social. Together, these dimensions constitute the legitimization basis for internalizing the new voice.

Technically, the genesis of vocal value is primarily anchored in self-perceivable competence growth. As students engage in sustained practice and exploration of the unfamiliar voice, they begin to register tangible improvements in their vocal mechanics: enhanced timbral stability, superior breath management, and refined phonatory precision. This somatic perception of technical advancement strips the unfamiliar voice of its status as a mere external pedagogical imposition, transmuting it into a tool of verifiable practical utility.

“*Firstly, I feel a tangible sense of personal progress. My voice sounds objectively better to my own ears; singing feels more comfortable, grounded, and sustainable over longer periods. My peers have also noticed this significant improvement. I believe my voice is continuously evolving in a trajectory that aligns with my future professional aspirations.” (S16)*

“*Previously, I relied entirely on shouting to reach high notes, while my teacher constantly emphasized bodily support. Now, my progress is substantial. My undergraduate peers tell me my voice is no longer as constricted or shrill as it used to be.” (S12)*

“*Compared to my freshman year, my vocal endurance has undeniably improved. I don't fatigue as easily when singing a complete aria, and my vocal range has slightly expanded.” (S18)*

Aesthetically, the value of the unfamiliar voice is established through the expansion of stylistic boundaries. Students progressively realize that the criteria for a “correct voice” are neither monolithic nor immutable; rather, they are contingent upon cultural, regional, stylistic, and even age-related variables. This epistemological shift fosters an open aesthetic disposition. Students cease fixating on a singular vocal standard, instead adopting a diverse lexicon—such as transparent, full-bodied, healthy, and natural—to redefine their internalized concept of the “correct voice.” This de-standardization and diversification of aesthetic criteria serves as a critical prerequisite for the unfamiliar voice to gain normative value.

“*Everyone's aesthetic perception of the ‘correct voice' varies. Some believe a forced, massive sound is desirable. Throughout my years of studying with my current teacher, my aesthetic paradigm has undergone a profound shift; I no longer consider a pushed sound to be a good sound.” (S11)*

“*Every individual possesses a unique aesthetic and a distinct vocal timbre. Every teacher belongs to a different pedagogical lineage. Therefore, there is simply no universally perfect definition of the ‘correct voice'.” (S15)*

Cognitively, value construction manifests as a reflective recalibration of the student's pre-existing vocal schema, extending beyond mere physical adaptation. Students begin to cross-reference evaluative standards across different pedagogues, utilizing this dialogic process to challenge and refine their initial vocal cognition. In essence, cognitive value is not directly transmitted by the teacher but is a product of continuous, self-authored reflection. As previously noted, S10—who was contradictorily classified by two different teachers—eventually utilized this cognitive dissonance to discover that a lyric soprano repertoire was her authentic vocal home. This sustained cognitive synthesis through interactions with multiple pedagogues was echoed by other participants:

“*In high school, my elderly teacher favored a very thin, bright aesthetic... In university, a younger teacher emphasized that contemporary folk aesthetics require a fusion of folk and bel canto elements... Now, in my graduate studies, my current teacher corroborates this, arguing that pure folk singing is less accepted by modern audiences, thus reinforcing the need for a bel canto foundation.” (S12)*

In terms of emotional value, the acceptance of the unfamiliar voice is characterized by progressive affective habituation. Through repeated practice and sustained exposure, students' initial aversion to the unfamiliar voice gradually dissipates, supplanted by an increasing sense of identification and emotional congruence. The initial somatic discomfort stabilizes into a sensation of comfort and expressive freedom.

“*Initially, I was profoundly unaccustomed to this sound. Later, as I sought to understand it, I gradually realized its merits, and eventually, I fell in love with it.” (S6)*

“*What truly facilitated my acceptance was the continuous experimentation and its resultant outcomes. When I discovered that the new voice enabled me to express emotions more effortlessly, I gradually validated it. With my teacher's guidance, I habituated to it, until I realized this voice had genuinely become a part of my own identity.” (S17)*

Socially, the legitimization of the unfamiliar voice is heavily fortified by external positive reinforcement. Affirmations from teachers, peer validation, and positive audience reception constitute vital external scaffolds. This social feedback, functioning synergistically with the preceding four dimensions, catalyzes the metamorphosis of the unfamiliar voice from a pedagogical mandate into a self-validated identity.

“*Initially, I obsessed over producing a heavy, dark tone, making my natural voice feel too thin and shrill. However, through continuous practice, I rediscovered my inherent voice rather than manufacturing an artificial one. Concurrently, peers and friends validated that this sound was clearer, brighter, and aesthetically superior, which allowed me to slowly accept it.” (S3)*

“*My teacher provides continuous feedback in the studio. When my voice meets her standards, she becomes visibly excited and offers profound affirmation.” (S6)*

Crucially, this overarching reconstruction of vocal value is reflected in the dynamic trajectory of students' self-evaluations. Rather than maintaining a static attitude toward their voices, students traverse a continuum from skepticism and negation to gradual acceptance and ownership. The realization that the “correct voice” must be an individualized construct rather than an external monolith represents a paramount outcome of this negotiation. Furthermore, the data suggest that these aesthetic and cognitive shifts are frequently precipitated by specific epiphanic events (e.g., studying abroad, transitioning to a new teacher) rather than occurring through a uniform, gradual accumulation. This indicates that vocal value reconstruction often exhibits a punctuated, stage-like trajectory triggered by critical environmental shifts.

“*I was accustomed to pure folk singing. When my new teacher demanded a fusion with bel canto, I was highly resistant. However, through relentless practice and aesthetic accumulation, I accepted my new voice, and I now find immense joy in singing with it.” (S12)*

“*My domestic teacher classified me as a fuller lyric tenor. However, upon studying abroad, my international teacher categorized me as a light lyric tenor. Recognizing that my coloratura agility was lacking, I eventually navigated back to my original vocal trajectory.” (S13)*

### 3.4 Developing vocal agency through autonomous decision-making

The development of vocal agency represents the most advanced stage of identity negotiation. At this stage, students no longer rely exclusively on their teachers' judgments but become capable of making independent vocal decisions based on their own values, experiences, and professional understanding. Rather than emerging as a single construct, vocal agency developed concurrently across three interrelated dimensions: evaluative judgment, competence, and self-efficacy.

The first manifestation of vocal agency was the reconstruction of evaluative judgment. Rather than relying on a single external authority, participants gradually developed more personalized and multidimensional criteria for evaluating vocal production. The “correct voice” was no longer determined solely by auditory perception or teacher feedback. Instead, students increasingly integrated bodily sensations, contextual factors, stylistic requirements, and their own listening experiences into a comprehensive evaluative framework.

Most participants reported drawing simultaneously upon multiple sources of information—including physical comfort, immediate auditory perception, recordings of their own performances, and teachers' feedback—to determine whether a vocal production was appropriate. This suggests that the development of vocal agency does not involve abandoning external guidance in favor of purely subjective intuition. Instead, students construct a more sophisticated evaluative system that integrates both internal and external sources of evidence.

“*When I encounter a new vocal sound, I first try to understand what that sound actually is. Then I pay attention to how my body is functioning, and I also listen to recordings of myself so that I can evaluate the sound more objectively. Through learning with my teacher, my aesthetic preferences have gradually become closer to my teacher's, and I often observe my teacher's lessons with other students to continue developing my own musical aesthetics.” (S5)*

“*I usually begin with my own bodily feelings—for example, whether singing feels smoother and more relaxed. I also listen to whether the resonance is stable. At the same time, I consider my teacher's reactions and feedback before making an overall judgment.” (S18)*

For some participants, this process extended beyond dependence on teacher authority. Teacher evaluations were no longer regarded as the sole criterion for judging vocal quality, and reliance on external evaluations gradually diminished. As a result, students developed relatively independent evaluative capacities, even when their own aesthetic judgments differed from those of their teachers.

“*My undergraduate teacher wanted me to sing with a much heavier sound, but it felt very uncomfortable, and it didn't feel like my own voice. I couldn't accept that sound because I knew that wasn't what my voice naturally sounded like—it was completely different from my speaking voice.” (S16)*

“*I communicate openly with my teacher because we have a very good relationship. However, there are some opinions that I simply don't agree with, and I'm comfortable accepting that difference.” (S7)*

Participants also described an increasingly embodied form of evaluation, in which proprioceptive and muscular sensations became reliable indicators of vocal quality. Rather than depending on external confirmation, bodily awareness itself became an important source of evaluative evidence:

“*Now I can judge and adjust my voice based on how my muscles feel. That's the result of years of practice. I no longer rely on feedback from my teacher or anyone else” (S12)*.

The second dimension of vocal agency was reflected in increased competence, characterized by greater flexibility in vocal production and enhanced self-regulation. With regard to vocal application, participants no longer regarded vocal production as conforming to a single ideal standard. Instead, they adjusted their voices according to the stylistic characteristics, emotional content, and expressive demands of different musical works. Appropriate vocal production therefore became context-dependent rather than universally defined.

“*Sometimes I adjust my voice according to the musical style. For lyrical songs and art songs I use one type of sound, while for more dramatic operatic arias I create a different vocal quality.” (S5)*

“*Now I actively think about how to adjust my voice according to the emotion and style of the music. If it's lyrical, I‘ll sing more gently; if it's more passionate, I'll add greater intensity. This has gradually become a habit through my teacher's guidance.” (S17)*

Competence was also demonstrated through increasingly autonomous self-regulation. Participants reported becoming more attentive to their physical condition and learning to modify their vocal strategies accordingly. As their ability to monitor vocal production improved, dependence on teacher feedback decreased, while sustainable bodily sensations became increasingly important as indicators of effective vocal technique.

“*When I couldn't distinguish vocal quality by listening, I depended heavily on my teacher. Later, once I developed my own listening ability, I was able to adjust my voice independently. Sometimes I even realised that the problem my teacher pointed out wasn't actually the real issue.” (S10)*

“*I first pay attention to my physical condition, then consider my teacher's feedback, and finally listen to whether the resonance is full and whether the tone matches what I want. I bring all of these factors together before deciding how to adjust my voice.” (S18)*

The third dimension of vocal agency was reflected in enhanced self-efficacy, extending beyond present learning to encompass confidence in future professional development. For some participants, confidence was closely linked to long-term career planning. They reported accepting unfamiliar vocal techniques because they recognized that developing greater vocal versatility would enhance future professional opportunities. Consequently, career aspirations became an active component of identity negotiation rather than merely an outcome of it.

“*Positive feedback from my classmates gives me confidence, and my career goals have helped me realise that mastering different vocal styles will broaden my future performance opportunities. Those long-term goals have encouraged me to embrace these changes in my voice.” (S17)*

“*With my teacher's continuous guidance, I've now been admitted to a doctoral programme. I feel that my voice is constantly developing, and that development fits well with my future career plans.” (S16)*

Self-efficacy was also reflected in participants' willingness and perceived ability to continue learning independently beyond formal education. Experiences of negotiating their vocal identities encouraged ongoing reflection and fostered confidence in managing future vocal development without continuous teacher supervision.

“*After graduation, I think I‘ll combine my teacher's advice with my own current situation. If I encounter something I don't understand, I'll still ask my teacher for guidance.” (S9)*

“*After these years of learning, I'm no longer as confused as I was at the beginning. I've grown a lot. I've learned to adjust myself more flexibly and to explore my own voice independently. I'll continue learning after graduation because I now believe I have the ability to manage my own vocal development.” (S18)*

## 4 Discussion

The findings illuminate how students gradually internalize the notion of the “correct voice” and negotiate their vocal identities within the context of one-to-one voice teaching. Rather than representing a linear process of technical acquisition, vocal identity development emerged as an ongoing negotiation between students' embodied experiences and the authority embodied in teachers' pedagogical discourse. This study demonstrates that students' uncertainty was rooted not simply in the difficulty of acquiring vocal techniques but, more fundamentally, in the ambiguity surrounding teachers' descriptions of the “correct voice.” Because vocal instruction frequently relies on subjective, metaphorical, and experience-based language, students were required to interpret meanings that were inherently open to multiple understandings.

Within this pedagogical context, students were not passive recipients of teacher authority. Instead, they continuously negotiated their relationships with that authority while attempting to reconcile teachers' expectations with their own bodily sensations, aesthetic preferences, and emerging professional identities. Consequently, teacher–student relationships extended beyond the simple transmission of knowledge and developed into complex interactions characterized by trust, dependence, skepticism, and critical reflection. Nevertheless, despite the diversity of these relational dynamics, the findings suggest that the establishment of a stable vocal identity ultimately depended not on the mechanical reproduction of authoritative standards but on the gradual development of students' agency and professional autonomy.

### 4.1 The ambiguity of the “correct voice”

One of the central findings of this study is that no universally shared standard exists for defining the “correct voice.” This ambiguity extends beyond pedagogical practice and is gradually internalized within students' own processes of meaning-making. Hausknecht et al. (2024), for example, identified 292 assessment descriptors across 64 higher education vocal curricula in English-speaking countries and demonstrated that approximately 61% of these terms could be eliminated through semantic consolidation. Their findings reveal substantial redundancy and ambiguity within the evaluative language employed by vocal teachers.

The present study similarly found that participants described the “correct voice” using highly individualized expressions such as resonant, full, healthy, and natural. However, the existence of multiple evaluative standards does not necessarily imply that students themselves possess vague conceptions of vocal quality. Rather, participants' uncertainty stemmed primarily from difficulties in interpreting the pedagogical language used by their teachers during one-to-one instruction.

A recurring finding across participants' accounts was that identical pedagogical terms frequently evoked different vocal images and auditory expectations for different individuals. Many participants repeatedly described teachers' explanations as “difficult to understand” and regarded vocal instruction as “highly abstract.” These accounts suggest that such difficulties are not isolated experiences but constitute a common characteristic of authentic one-to-one voice teaching contexts. This finding is consistent with the experimental work of Vurma and Ross (2002), who demonstrated that even experienced voice teachers were unable to consistently distinguish the vocal characteristics represented by widely used pedagogical terminology based solely on auditory perception. If experienced teachers themselves cannot consistently agree on the auditory referents of these descriptors, students' uncertainty should not be interpreted as a deficiency in learning ability. Instead, it reflects the inherently indeterminate nature of the pedagogical language itself.

Whereas Vurma and Ross (2002) focused on the instability of individual vocal descriptors under experimental conditions, the present findings suggest that such ambiguity extends beyond isolated terminology and characterizes metaphor-based vocal instruction more broadly. This observation contributes new empirical evidence from authentic teaching contexts to the continuing debate surrounding imagery-based vocal pedagogy.

Recent scholarship presents contrasting perspectives on this issue. Brown (2025) argues that clean language and imagery-based instruction, grounded in teachers' embodied professional experience, facilitates students' acquisition of expert knowledge. In contrast, Lillis (2021), Henrich et al. (2008), Morales-Villar et al. (2023), and Ware (2013) contend that metaphorical instructional language may generate misunderstandings and constrain students' vocal development. The findings of the present study align more closely with the latter perspective. Participants consistently associated learning difficulties with metaphorical and image-based verbal instruction, whereas demonstrations and embodied forms of guidance were rarely identified as sources of confusion.

Beyond the characteristics of instructional language itself, structural differences in professional expertise between teachers and students may also contribute to tensions arising from aesthetic disagreement and divergent perceptions of vocal production. Frič and Pavlechová (2020) demonstrated that experienced voice teachers exhibited significantly greater agreement and accuracy than non-specialist musicians when classifying vocal voice types, suggesting that teachers' evaluative judgments are generally more reliable because of their accumulated professional expertise. Students, however, have not yet developed similarly stable evaluative frameworks. Consequently, when their own vocal perceptions conflict with those of their teachers, they often struggle to determine whether such discrepancies reflect differences in aesthetic preference or limitations in their own developing evaluative capacity. The ambiguity surrounding the “correct voice” therefore arises not only from the indeterminate nature of pedagogical language but also from the asymmetrical distribution of professional knowledge and evaluative expertise within one-to-one voice teaching.

### 4.2 Trust, tension, and strategic compliance in teacher–student relationships

The starting point of vocal identity negotiation lies in the perceived discrepancy between students' existing vocal production and the “correct voice” expected by teachers. However, because the “correct voice” lacks a stable or universally agreed-upon definition and is frequently articulated through ambiguous evaluative terminology, this discrepancy is not easily interpreted or resolved by students independently. Instead, it must be negotiated within the relational dynamics of one-to-one voice teaching.

The tensions observed in this study are therefore grounded in the structural characteristics of one-to-one pedagogy itself, where teachers occupy a position of continuous presence and high-frequency evaluation. Gaunt (2008) conceptualizes the voice teacher in one-to-one contexts as a value-bearing authority with asymmetrical power, while Johansson (2013) suggests that teacher–student interaction is a mutually constitutive process of influence and response. Similarly, Crocco et al. (2020) highlight the unusually high frequency of evaluative feedback in one-to-one voice teaching. Together, these studies provide a conceptual foundation for understanding the structural conditions underpinning teacher–student interaction in vocal pedagogy.

Existing literature has often implied a contrast between competitive conservatoire environments and the presumed relational stability of one-to-one instruction. Kingsbury (1988), for instance, describes conservatoire settings as highly competitive environments that may negatively affect students' self-esteem. In contrast, Fletcher et al. (2024) suggest that one-to-one teacher–student relationships are typically characterized by trust and are less likely to generate such adverse effects. The findings of the present study complicate this binary framing. Interview data revealed frequent expressions of trust and recognition of teachers' authority; however, expressions of disagreement and mistrust were also evident. This suggests that one-to-one voice teaching should not be conceptualized as a uniformly trusting relational space, but rather as a dynamic field in which trust and skepticism coexist.

One possible explanation for this divergence lies in the structural heterogeneity of the sample. Whereas previous studies often compare institutional environments (e.g., conservatoire ensembles vs. one-to-one instruction), the present study draws on participants with diverse backgrounds in terms of vocal style, educational level, and international study experience. Such heterogeneity may have contributed to a more differentiated and less homogeneous representation of teacher–student relationships.

At the same time, the presence of strong trust in teachers raises an important question: to what extent might high levels of reliance on a single teacher constrain the development of student autonomy? Gaunt (2010), Black (2017), and Harrison and Grant (2015) argue that excessive dependence on a single teacher's judgement may limit the development of learner autonomy and weaken long-term self-directed learning capacities. The present findings suggest that evaluative judgment among students is not uniform but instead develops along a continuum. Some students rely primarily on teacher judgement with limited independent evaluative frameworks; others integrate bodily sensations with teacher feedback to construct more diversified reference systems; and a further group demonstrates relatively stable independent evaluative criteria, at times maintaining their own aesthetic judgments and engaging in proactive self-regulation. This gradation indicates that dependence and independence should not be understood as a binary opposition, but rather as a spectrum of evolving evaluative agency.

Beyond its implications for autonomy development, relational tension in teacher–student interaction also manifests in immediate emotional and behavioral responses within instructional settings. Gaunt (2011) notes that strained pedagogical relationships may evoke anxiety, avoidance, or resistance in students. The present study identifies an additional and less frequently theorized response: strategic compliance. Rather than openly resisting or withdrawing, students may outwardly conform to teachers' instructions while internally maintaining their own evaluative stance. This form of compliance is neither genuine acceptance nor explicit resistance; instead, it constitutes an intermediate, strategic mode of negotiation that warrants conceptual recognition in its own right.

This finding resonates with the quantitative study by Blackwell et al. (2025), which examined 151 music students and found that when students sought teacher approval or attempted to avoid rejection, their motivation shifted from intrinsic to extrinsic orientations, with increased sensitivity to teacher evaluation rather than personal progress. Such strategic compliance may be closely linked to the structural dependence of one-to-one voice teaching on immediate, directive forms of feedback. This pedagogical structure constrains opportunities for independent exploration, thereby making surface-level compliance combined with internal reservation a pragmatic strategy for navigating high-density instructional environments.

While autonomy-supportive pedagogies have been argued to enhance student wellbeing (Bonneville-Roussy et al., 2020), and effective teaching has been conceptualized as fostering independence (Chapman, 2006), the present findings suggest that such approaches are not consistently reflected in everyday vocal instruction. Instead, consistent with Crocco et al. (2020), control-oriented feedback and demonstration remain dominant pedagogical practices in one-to-one voice teaching, whereas strategies associated with self-monitoring, motivational scaffolding, and perceptual training appear comparatively less frequent. Although high-frequency directive instruction may be pedagogically effective, its unintended consequence may be the compression of students' opportunities for autonomous exploration.

Finally, the emotionally charged nature of this instructional context is amplified by the intimate coupling of voice, body, and identity. Lewis and Hendricks (2022) argue that, in the absence of an external instrument as a mediating buffer, corrective feedback in vocal learning is more likely to be perceived as personal criticism, with negative evaluations potentially having a profound impact on students' self-concept. Vocal change therefore extends beyond technical modification, directly implicating processes of self-evaluation and identity construction. This perspective helps explain both the intensity of students' emotional experiences observed in this study and the emergence of strategic compliance as a protective mechanism. Through outward conformity, students may reduce exposure to potentially destabilizing feedback while preserving an internal space for independent evaluation and identity negotiation.

### 4.3 The significance of autonomy in the formation of vocal identity

The outcome of vocal identity negotiation is most clearly reflected in the emergence of vocal agency. At this stage, students no longer rely exclusively on teachers' judgments but are able to make independent vocal decisions grounded in their own values and embodied experience across three interrelated dimensions: evaluative judgment, competence, and self-efficacy.

This finding also addresses a longstanding gap in vocal identity research. Crow et al. (2021) argue that congruence between voice and self-concept is central to understanding identity formation. However, limited systematic attention has been paid to the processes through which such congruence emerges, is disrupted, and is subsequently reconstructed. The present study suggests that this congruence develops through two distinct pathways: one based on identification with and trust in teacher authority, and another based on autonomous construction. The latter directly reflects the development of vocal agency, whereby students, through their evolving evaluative and performative capacities, are able to independently assess, adjust, and ultimately affirm that “this voice is my voice,” without reliance on external authority.

At the same time, a substantial number of students reported a transition from initial non-recognition of their own voice to eventual acceptance. This shift was facilitated by identifiable experiential turning points, including sustained practice, changes in aesthetic orientation shaped by contextual exposure, and broader affective or attitudinal transformations. These findings indicate that congruence between voice and self-concept is not achieved instantaneously; rather, it involves an initial phase of discomfort and misrecognition, followed by gradual reconstruction through specific experiential catalysts. In this sense, the present study extends Crow et al.'s (2021) discussion by empirically illustrating how voice–self congruence is both disrupted and reconstructed over time.

Vocal agency is first manifested in the dimension of evaluative judgment. Whether students are able to follow a pathway of “self-construction” depends on their capacity to evaluate what constitutes a “correct voice.” Students who lack such capacity are more likely to rely on teacher identification to achieve congruence, whereas those who develop evaluative agency are able to independently assess whether a sound “matches who I am” and construct their own evaluative criteria accordingly. Participants' evaluative development thus revealed a continuum ranging from teacher dependence, to the integration of multiple referential systems, and finally to relatively independent judgment. This gradient resonates with Després and Dubé (2020) spectrum of learner participation in educational decision-making.

The second dimension of vocal agency is competence, defined as the ability to flexibly deploy vocal resources across contexts and to continuously monitor and regulate one's own vocal production. Lewis and Hendricks (2022) found that vocal learners gradually develop the capacity for independent and reflective self-assessment, which in turn enhances self-corrective ability. The findings of the present study closely align with this mechanism. Participants no longer relied solely on immediate teacher feedback to determine vocal appropriateness; instead, they actively attended to bodily states and adjusted vocal production in relation to stylistic demands, emotional content, and performance goals. This shift indicates that students progressively assume responsibility for real-time monitoring and adjustment of vocal output, a function traditionally occupied by the teacher, thereby extending autonomy from judgment to action.

The third dimension of vocal agency, self-efficacy, extends the outcomes of negotiation into students' orientations toward their future trajectories, constituting a further layer of identity resolution identified in this study. Some participants explicitly reported having future-oriented plans and perceiving certain vocal qualities as aligned with their professional aspirations. However, the relationship between future orientation and identity resolution is not unidirectional. In some cases, it emerges as a natural extension of already established evaluative coherence; in others, it functions as an anticipatory rationale that enables students to accept present vocal demands. In such cases, congruence is retrospectively constructed after the decision to accept a particular vocal orientation has already been made.

The centrality of vocal agency in identity negotiation is further supported by existing literature. Previous studies have consistently demonstrated that vocal agency is a key determinant of the stability of vocal identity. Hogle (2021), for instance, shows that when individuals' singing agency is undermined through negative evaluative experiences, this disruption is often internalized as a stable self-negation, leading to avoidance behaviors and protective strategies such as concealing one's voice. In such cases, vocal identity becomes characterized by persistent shame and instability. Conversely, the loss of agency directly contributes to the fragmentation of vocal identity. In a complementary direction, Sweet and Parker (2019) define autonomy as the capacity to govern, revise, and develop one's own musical growth, conceptualizing it as a central developmental goal in emerging adulthood. They further argue that, compared with instrumentalists, vocal learners are more likely to develop autonomy under conditions of heightened exposure, due to the absence of an external mediating instrument and the intrinsic coupling of voice and body. Such autonomy is therefore central to the establishment of stable vocal identity. Taken together, these studies converge on the conclusion that autonomy, rather than specific technical proficiency or normative vocal standards, is fundamental to the stability of vocal identity.

The mechanism through which vocal agency emerges can also be situated within broader theories of identity formation. Monks (2003), Parker (2014), Després and Dubé (2020), Forbes et al. (2025), Chong et al. (2024), and Hogle (2021) collectively argue that vocal identity is neither an isolated cognitive construct nor a passive internalization of external evaluation. Rather, it is a dynamic process that integrates bodily perception, self-monitoring, prior experience, and social evaluation. In particular, Forbes et al. (2025) four identity principles—distinctiveness, self-esteem, self-efficacy, and continuity—highlight self-efficacy as a central driver of identity construction and maintenance. The present study's findings regarding students' confidence in future development align closely with this principle.

Empirically, participants reported that vocal judgments were made by integrating bodily comfort, musical interpretation, recorded self-listening, and teacher feedback. This provides concrete evidence for an integrative model of identity formation, in which evaluative judgment and competence are developed through the incorporation of increasingly diverse referential systems rather than the replacement of one source of authority by another. Accordingly, the three dimensions of evaluative judgment, competence, and self-efficacy collectively suggest that vocal identity negotiation does not culminate in a simple determination of vocal correctness. Instead, it reflects a progressive process through which students acquire increasing autonomy in judging, using, and developing their voices. This process constitutes both the mechanism through which voice–self congruence is reconstructed and the defining outcome of identity negotiation itself.

## 5 Conclusion

The core contribution of this study lies in addressing a long-standing gap in the literature on student vocal identity: how congruence between the voice and self-perception is formed. Over time, students perceive and conceptualize the idealized “correct” voice through their teachers' subjective descriptions and evaluations. They develop a degree of identification with this voice within the teacher-student relationship and continually reflect upon it amidst the uncertainties of practice. If students can cultivate an autonomous value judgment regarding the “correct” voice, successful identity negotiation can be achieved. In this context, “negotiation” is not merely a process of passively accepting or resisting teacher authority; rather, it is a concretely delineable psychological process jointly constructed by cognitive capacity and a sense of congruence.

Consequently, these results carry profound implications for vocal pedagogy, advocating a shift from authoritative instruction to an autonomy-supportive paradigm. Rather than an imposed endpoint, the “correct voice” emerges as a developmental trajectory. To navigate this, students must operationalize their agency by reframing cognitive dissonance as a normative part of learning, utilizing somatic feedback for self-verification, and claiming ownership of their evaluative judgments. Concurrently, educators should provide targeted pedagogical scaffolding. This entails replacing ambiguous metaphors with explicit auditory modeling, allowing temporal space for vocal exploration without hyper-correction, and systematically transferring evaluative responsibility to the student. Ultimately, by delivering mechanics-focused feedback that separates the voice from the ego, educators can foster profound somatic congruence and safeguard students' developing vocal identities.

## Data Availability

The original contributions presented in the study are included in the article/supplementary material, further inquiries can be directed to the corresponding author.
